# Supplementary material for: A Critical View of the Application of the APEX Software (Aqueous Photochemistry of Environmentally-Occurring Xenobiotics) to Predict Photoreaction Kinetics in Surface Freshwaters
Source: Molecules. 2019 Dec 18;25(1):9. doi: 10.3390/molecules25010009 (PMC7017383; doi:10.3390/molecules25010009)
Supplement: Supplementary file 1 [file molecules-25-00009-s001.zip › 3.2.4_gcc-4.4.0.zip!/3.2.4_gcc-4.4.0/3.2.4_gcc-4.4.0/share/octave/packages/nnet-0.1.10/doc/pdf/neuralNetworkPackageForOctaveUsersGuide.pdf]

A neural network package for Octave
User's Guide
Version: 0.1.9.1

Michel D. Schmid

January 15, 2009

Contents

1 Introduction **2**
1.1 Compatibility to Matlab's ^{TM}Neural Network Toolbox . . . 2
1.2 Version numbers . . . 2
1.3 Known incompatibilities . . . 2
1.3.1 Function names . . . 2

2 Neural Network Package for Octave **3**
2.1 Available Functions . . . 3
2.1.1 min_max . . . 3
2.1.2 newff . . . 3
2.1.3 prestd . . . 4
2.1.4 poststd . . . 4
2.1.5 saveMLPStruct . . . 4
2.1.6 sim . . . 4
2.1.7 subset . . . 4
2.1.8 train . . . 5
2.1.9 trastd . . . 6
2.2 Transfer functions . . . 6
2.2.1 logsig . . . 6
2.2.2 purelin . . . 6
2.2.3 tansig . . . 7

3 Examples **8**
3.1 Example 1 . . . 8
3.1.1 Introduction . . . 8
3.1.2 Code m-file . . . 8
3.1.3 Walkthrough . . . 10
3.2 Example 2 . . . 11
3.2.1 Introduction . . . 11
3.2.2 Code m-file . . . 11
3.2.3 Walkthrough . . . 13

Chapter 1

Introduction

1.1 Compatibility to Matlab's ^{TM}Neural Network Toolbox

The compatibility is one of the strongest targets during developing this toolbox. If I have to develope an incompatibility e.g. in naming the functions, it will be descriped in this documentation. Even though it should be clear that I can't make a one to one copy. First, the m-files are copyrighted and second, octave doesn't yet support the object oriented-programming techonology.

If you find a bug, any not described incompatibility or have some suggestions, please write me at michaelschmid@users.sourceforge.net. This will help improving this toolbox.

1.2 Version numbers

The first number describes the major release. Version number V1.0 will be the first toolbox release which should have the same functions like the Matlab R14 SP3 neural network Toolbox.

The second number defines the finished functions. So to start, only the MLPs will realised and so this will be the number V0.1.0.

The third number defines the status of the actual development and function. V0.1.0 means a first release with MLP. Actually it works only with Levenberg-Marquardt algorithm and Mean-Square-Error as performance function.

1.3 Known incompatibilities

1.3.1 Function names

minmax

minmax is in this toolbox called *min_max*. This is because Octave already has a function whichs name is *minmax*. This is a c file and the functions *min* and *max* are therein realized.

Chapter 2

Neural Network Package for Octave

This chapter describes all functions available in the neural network package of Octave.
Eventhough it will be as compatible as possible to the one of MATLAB(TM).

2.1 Available Functions

2.1.1 min_max

min_max get the minimal and maximal values of an training input matrix. So the dimension of this matrix must be an RxN matrix where R is the number of input neurons and N depends on the number of training sets.

Syntax:

mMinMaxElements = min_max(RxN);

Description:

RxN: R x N matrix of min and max values for R input elements with N columns

Example:

$$\begin{bmatrix} 1 & 11 \\ 0 & 21 \end{bmatrix} = min\_max \begin{bmatrix} 3 & 1 & 3 & 5 & 11 \\ 12 & 0 & 21 & 8 & 6 \end{bmatrix} \tag{2.1}$$

2.1.2 newff

newff is the short form for ***new feed forward network***. This command creates a feed-forward backpropagation network structure.

Syntax:

net = newff(Rx2,[S1 S2 ... SN],{TF1 TF2 ... TFN},BTF,BLF,PF)

Description:

Rx2: R x 2 matrix of min and max values for R input elements
Si: Size of ith layer, for N layers
TFi: Transfer function of ith layer, default = "tansig"
BTF: Backpropagation network training function, default = "trainlm"
BLF: Backpropagation weight/bias learning function, NOT USED, is only for MATLAB(TM) compatibility

PF: Performance function, default = "mse"

Examples:

net = newff(Rx2,[2 1])
net = newff(Rx2,[2 1],{"tansig","purelin"});
net = newff(Rx2,[2 1],{"tansig","purelin"},"trainlm");
net = newff(Rx2,[2 1],{"tansig","purelin"},"trainlm","notUsed","mse");

Comments:
In this version, you can have as much output neurons as you want. The same with the number of hidden layers. This means you can have one input layer, unrestricted number of hidden layers and one output layer. That's it.

2.1.3 prestd

2.1.4 poststd

2.1.5 saveMLPStruct

This is an additional function which doesn't exist in the neural network toolbox of MathWorks (TM). To see the network structure, you can use this command and save the complete structure to a file. Open this file and you have the same view like you would open the *network type* of MATLAB(TM).

Syntax:

saveMLPStruct(net,"initNetwork.txt");

2.1.6 sim

Syntax:

simout = sim(net,P);

Description:

Left-Hand-Side:
simout: Output values of the simulated network.

Right-Hand-Side:
net: Network, created and trained with newff(...) and train(...)
P: Input data

2.1.7 subset

subset can be used to optimize the data sets for train, test and validation of a neural network.

Syntax:

[mTrain, mTest, mVali] = subset(mData,nTargets,iOpti,fTest,fVali);

Description:

Left-Hand-Side:
mTrain: (R+T) x M matrix with R input rows, T output rows and M columns where M <= N.
mTest: (R+T) x S matrix with R input rows, T output rows and S columns where S <= N.
mVali: (R+T) x U matrix with R input rows, T output rows and U columns where U <= N. And U can only exist, if S also exist.

Right-Hand-Side:
mData: (R+T) x N matrix with R input rows, T output rows and N columns
nTargets: Number of T output rows
iOpti: Integer value to define level of optimization.
fTest: Fraction to define the percentage of data sets which should be used for testing.
fVali: Fraction to define the percentage of data sets which should be used for testing.

iOpti can have following values:
0 : no optimization
1 : will randomise the column order and rerange the columns containing min and max values to be in the train set
2 : will NOT randomise the column order, but rerange the columns containing min and max values to be in the train set

fTest or fValie have following meaning:
Each of this arguments can be a fraction or zero. The value 1 is not allowed! The sum of both values must also be smaller than 1!
Example: fTest = 1/3

Default values
iOpti = 1
fTest = 1/3
fVali = 1/6

Examples:

mTrain = subset(mData,2,1,0,0)
[mTrain,mTest] = subset(mData,2,1,1/3,0);
[mTrain,mTest,mVali] = subset(mData,1);
[mTrain,mTest,mVali] = subset(mData,1,1,1/3,1/6);

2.1.8 train

Syntax:

net = train(MLPnet,P,T,[],[],VV);

Description:

Left-Hand-Side:
net: Trained multi-layer network.

Right-Hand-Side:
MLPnet: Multi-layer network, created with newff(...)
P: Input data for training

T: Target data for training
[]: Not used right now, only for compatibility with Matlab
[]: Not used right now, only for compatibility with Matlab
VV: Validation data. Contains input and target values

Examples:

net = train(MLPnet,P,T)
net = train(MLPnet,P,T,[],[],VV)

P,T must have the same number of rows as in newff(...).
VV.P, VV.T must have the same number of rows as in newff(...)

Comments:
Please be sure to put the validation values in a structure named VV.P and VV.T.
VV can be changed, but not .P and .T!

2.1.9 trastd

2.2 Transfer functions

2.2.1 logsig

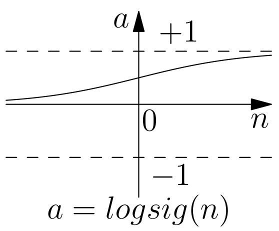

Figure 2.1: Log-Sigmoid transfer function

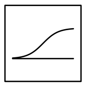

Figure 2.2: Log-Sigmoid transfer function logo

2.2.2 purelin

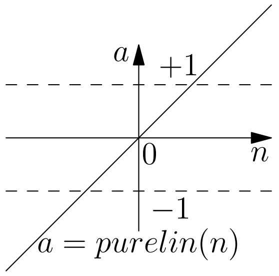

Figure 2.3: Linear transfer function

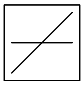

Figure 2.4: Linear transfer function logo

2.2.3 tansig

I solved all of my real life problems with this transfer function if a non-linear function was used. In [4] page 2-6 the tansig is defined as in equation (2.2). A look on the MathWorks homepage with the keyword tansig will show that tansig is programed as in equation (2.3).

$$a = \frac{e^n - e^{-n}}{e^n + e^{-n}} \tag{2.2}$$

$$a = \frac{2}{(1 + e^{-2*n}) - 1} \tag{2.3}$$

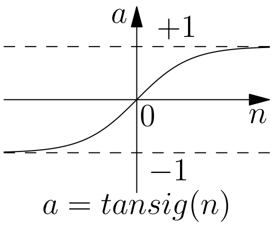

Figure 2.5: Tansig transfer function

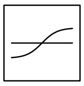

Figure 2.6: Tansig transfer function logo

Chapter 3

Examples

3.1 Example 1

You can find this example in the *tests/MLP* directory of each release or from the subversion repository. I will do (more or less) a line by line walkthrough, so after this should be everything clear. I assume that you have some experience with multilayer perceptrons.

3.1.1 Introduction

Our problem can be solved with a monotonically increasing or decreasing surface. An input vector **p** (with 9 values) should be mapped onto one output value. Because we know that it can be solved with a monotonically increasing or decreasing surface, we can choose a 9-1-1 multi-layer perceptron (short: MLP). This means an MLP with 9 input neurons, only 1 hidden neuron and with 1 output neuron.

3.1.2 Code m-file

```
## Copyright (C) 2006 Michel D. Schmid  <michaelschmid@users.sourceforge.net>
##
##
## This program is free software; you can redistribute it and/or modify it
## under the terms of the GNU General Public License as published by
## the Free Software Foundation; either version 2, or (at your option)
## any later version.
##
## This program is distributed in the hope that it will be useful, but
## WITHOUT ANY WARRANTY; without even the implied warranty of
## MERCHANTABILITY or FITNESS FOR A PARTICULAR PURPOSE.  See the GNU
## General Public License for more details.
##
## You should have received a copy of the GNU General Public License
## along with this program; see the file COPYING.  If not, write to the Free
## Software Foundation, Inc., 51 Franklin Street, Fifth Floor, Boston, MA
## 02110-1301, USA.

## Author:  Michel D. Schmid


## load data
mData = load("mData.txt","mData");
mData = mData.mData;
```

```
[nRows, nColumns] = size(mData);
    # this file contains 13 columns.
    # The first 12 columns are the inputs
    # the last column is the output,
    # remove column 4, 8 and 12!
    # 89 rows.
mOutput = mData(:,end);
mInput = mData(:,1:end-1);
mInput(:,[4 8 12]) = []; # delete column 4, 8 and 12
## now prepare data
mInput = mInput';
mOutput = mOutput';
# now split the data matrix in 3 pieces, train data, test data and validate data
# the proportion should be about 1/2 train, 1/3 test and 1/6 validate data
# in this neural network we have 12 weights, for each weight at least 3 train sets..
# (that's a rule of thumb like 1/2, 1/3 and 1/6)
# 1/2 of 89 = 44.5; let's take 44 for training
nTrainSets = floor(nRows/2);
# now the rest of the sets are again 100%
# ==> 2/3 for test sets and 1/3 for validate sets
nTestSets = (nRows-nTrainSets)/3*2;
nValiSets = nRows-nTrainSets-nTestSets;
mValiInput = mInput(:,1:nValiSets);
mValliOutput = mOutput(:,1:nValiSets);
mInput(:,1:nValiSets) = [];
mOutput(:,1:nValiSets) = [];
mTestInput = mInput(:,1:nTestSets);
mTestOutput = mOutput(:,1:nTestSets);
mInput(:,1:nTestSets) = [];
mOutput(:,1:nTestSets) = [];
mTrainInput = mInput(:,1:nTrainSets);
mTrainOutput = mOutput(:,1:nTrainSets);
[mTrainInputN,cMeanInput,cStdInput] = prestd(mTrainInput);# standardize inputs
## comments:  there is no reason to standardize the outputs because we have only
# one output ...
# define the max and min inputs for each row
mMinMaxElements = min_max(mTrainInputN); # input matrix with (R x 2)...
## define network
nHiddenNeurons = 1;
nOutputNeurons = 1;
MLPnet = newff(mMinMaxElements,[nHiddenNeurons nOutputNeurons],\
        {"tansig","purelin"},"trainlm","","mse");
## for test purpose, define weights by hand
MLPnet.IW{1,1}(:)  = 1.5;
```

```
00079 MLPnet.LW{2,1}(:)  = 0.5;
00080 MLPnet.b{1,1}(:)  = 1.5;
00081 MLPnet.b{2,1}(:)  = 0.5;
00082
00083 saveMLPStruct(MLPnet,"MLP3test.txt");
00084
00085 ## define validation data new, for matlab compatibility
00086 VV.P = mValiInput;
00087 VV.T = mValliOutput;
00088
00089 ## standardize also the validate data
00090 VV.P = trastd(VV.P,cMeanInput,cStdInput);
00091
00092 [net] = train(MLPnet,mTrainInputN,mTrainOutput,[],[],VV);
00093
00094 # make preparations for net test and test MLPnet
00095 #  standardise input & output test data
00096 [mTestInputN] = trastd(mTestInput,cMeanInput,cStdInput);
00097
00098 [simOut] = sim(net,mTestInputN);
00099 simOut
```

3.1.3 Walkthrough

Till line number 0023 there is realy nothing interesting.
On line 0023 & 0024 data will be loaded. This data matrix contains 13 columns. Column 4, 8 and 12 won't be used (this is because the datas are of a real world problem). Column 13 contains the target values. So on the lines 0049 till 0051 this will be splittet into the corresponding peaces. A short repetition about the datas: Each line is a data set with 9 input values and one target value. On line 0038 and 0039 the datas are transposed. So we have now in each column one data set.

Now let's split the data matrix again in 3 pieces. The biggest part is for training the network. The second part for testing the trained network to be sure it's still possible to generalize with the net. And the third part, and the smallest one, for validate during training. This splitting happens on the lines 0041 till 0061.

Line 0063 is the first special command from this toolbox. This command will be used to pre-standardize the input datas. Do it ever! Non linear transfer functions will squash the whole input range to an small second range e.g. the transfer function *tansig* will squash the datas between -1 and +1.

On line 0069 the next toolbox command will be used. This command *min_max* creates a $Rx2$ matrix of the complete input matrix. Don't ask me for what MATLAB(TM) this is using. I couldn't figure out it. One part is the number of input neurons, but for this, the range would not be needed. Who cares ;-)

Now it's time to create a structure which holds the informations about the neural network. The command **newff** can do it for us. See the complete line and actually, please use it only on this way, each other try will fail! This means, you can change the number of input neurons, the number of hidden neurons and the number of output neurons of course. But don't change the train algorithm or the performance function.

saveMLPStruct on line 0083 is a command which doesn't exist in MATLAB(TM). This will save the structure with the same informations you can see in MATLAB(TM) if you try to open the net-type.

The validation part on line 0086 & 0087 is important. The naming convention is for MATLAB(TM) compatibility. For validate, you have to define a structure with the name **VV**. Inside this structure you

have to define actually **VV.P** & **VV.T** for validate inputs and validate targets. Bye the way, you have to pre-standardize them like the training input matrix. Use for this the command **trastd** like on line 0090.

train is the next toolbox command and of course one of the most important. Please also use this command like on line 0092. Nothing else will work.

The second last step is to standardize again datas. This time the test datas. See line 0096 for this and the last step. Simulate the network. This can be done with the command **sim**. This will be a critical part if someone else will write a toolbox with this command name!

I hope this short walkthrough will help for first steps. In next time, I will try to improve this documentation and of course, the toolbox commands. But time is realy rare.

3.2 Example 2

You can find this example in this directory but renamed to *MLP9_1_1.m_template*. I will explain only differencies to the example1, so please read it first, if you haven't.

3.2.1 Introduction

Our problem can be solved with a monotonically increasing or decreasing surface. An input vector **p** (with 9 values) should be mapped onto one output value. Because we know that it can be solved with a monotonically increasing or decreasing surface, we can choose a 9-1-1 multi-layer perceptron (short: MLP). This means an MLP with 9 input neurons, only 1 hidden neuron and with 1 output neuron.

3.2.2 Code m-file

```
## Copyright (C) 2008 Michel D. Schmid  <michaelschmid@users.sourceforge.net>
##
##
##
## This program is free software;you can redistribute it and/or modify it
## under the terms of the GNU General Public License as published by
## the Free Software Foundation; either version 2, or (at your option)
## any later version.
##
## This program is distributed in the hope that it will be useful, but
## WITHOUT ANY WARRANTY; without even the implied warranty of
## MERCHANTABILITY or FITNESS FOR A PARTICULAR PURPOSE.  See the GNU
## General Public License for more details.
##
## You should have received a copy of the GNU General Public License
## along with this program; see the file COPYING.  If not, see
## http://www.gnu.org/licenses.

## Author:  Michel D. Schmid


## load data
mData = load("mData.txt","mData");
mData = mData.mData;
[nRows, nColumns] = size(mData);
    # this file contains 13 columns.
    # The first 12 columns are the inputs
```

```
    # the last column is the output,
    # remove column 4, 8 and 12!
    # 89 rows.

mOutput = mData(:,end);
mInput = mData(:,1:end-1);
mInput(:,[4 8 12]) = []; # delete column 4, 8 and 12

mData = [mInput mOutput];

# now split the data matrix in 3 pieces, train data, test data and validate
data
# the proportion should be about 1/2 train, 1/3 test and 1/6 validate data
# in this neural network we have 12 weights, for each weight at least 3
train sets..
# that's a rule of thumb like 1/2, 1/3 and 1/6
# 1/2 of 89 = 44.5; let's take 44 for training
[mTrain,mTest,mVali] = subset(mData',1);

[mTrainInputN,cMeanInput,cStdInput] = prestd(mTrain(1:end-1,:));
                                      #standardize inputs

## comments:  there is no reason to standardize the outputs because we have
only
# one output ...

# define the max and min inputs for each row
mMinMaxElements = min_max(mTrainInputN); # input matrix with (R x 2)...

## define network
nHiddenNeurons = 1;
nOutputNeurons = 1;

MLPnet = newff(mMinMaxElements,[nHiddenNeurons nOutputNeurons],\
        {"tansig","purelin"},"trainlm","","mse");
## for test purpose, define weights by hand
MLPnet.IW{1,1}(:)  = 1.5;
MLPnet.LW{2,1}(:)  = 0.5;
MLPnet.b{1,1}(:)  = 1.5;
MLPnet.b{2,1}(:)  = 0.5;

saveMLPStruct(MLPnet,"MLP3test.txt");

## define validation data new, for matlab compatibility
VV.P = mVali(1:end-1,:);
VV.T = mVali(end,:);

## standardize also the validate data
VV.P = trastd(VV.P,cMeanInput,cStdInput);

[net] = train(MLPnet,mTrainInputN,mTrain(end,:),[],[],VV);

# make preparations for net test and test MLPnet
#  standardise input & output test data
```

```
00082 [mTestInputN] = trastd(mTest(1:end-1,:),cMeanInput,cStdInput);
00083
00084 [simOut] = sim(net,mTestInputN);
00085 simOut
```

3.2.3 Walkthrough

The difference to the example1 starts below the line number 0035.
The difference concerns only the pre-processing section where the data set is splittet into the three subsets. This time, the command **subset** is used, which makes the complete example about 19 lines shorter! On line 0023 & 0024 data will be loaded. This data matrix contains 13 columns. Column 4, 8 and 12 won't be used (this is because the datas are of a real world problem). Column 13 contains the target values.

Now on line 35 we have to merge the input and output targets again. Subset will take the complete matrix as argument! On line 42 happens the complete magic :-). Subset will return three subsets containing each time the input and output arguments. So this part must be splitet once more! But this is very easy and happens at some specific positions below.

That's it, **subset** will help you to write short scripts!

Bibliography

[1] John W. Eaton

GNU Octave Manual, Edition 3, PDF-Version, February 1997

[2] The MathWorks, Inc.

MATLAB Help, MATLAB Version 7.1 (R14SP3), Neural Network Toolbox Version 4.0.6 (R14SP3)

[3] Christopher M. Bishop

Neural Networks for Pattern Recognition, OXFORD University Press, Great Clarendon Streed, Oxford OX2 6DP, ISBN 0-19-853864-2, 2002

[4] Martin T. Hagen, Howard B. Demuth, Mark H. Beale

NEURAL NETWORK DESIGN, PWS Publishing Company, 20 Park Plaza, Boston, MA 02116-4324, ISBN 053494332-2, 1996
